# Supplementary material for: A unified approach for quantifying and interpreting DNA shape readout by transcription factors
Source: Mol Syst Biol. 2018 Feb 22;14(2):e7902. doi: 10.15252/msb.20177902 (PMC5822049; doi:10.15252/msb.20177902)
Supplement: Supplementary file 1 — Expanded View Figures PDF [file MSB-14-e7902-s001.pdf]

Expanded View Figures

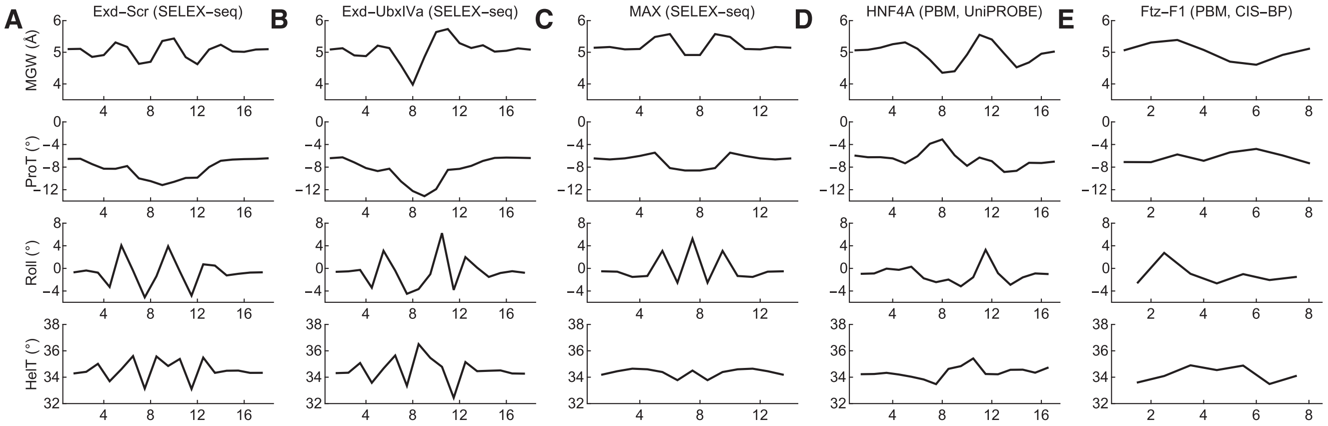

Figure EV1. Shape profiles of high-affinity SELEX and PBM probes.

A Mean shape of Exd-Scr SELEX-seq probes with relative affinity greater than 0.1. The probes were aligned to match Fig 2A and B (see Materials and Methods).

B, C The same as (A) but showing Exd-UbxIVa and MAX.

D Mean shape of affinity HNF4A PBM probes (Robasky & Bulyk, 2011). The top 100 probes were used.

E The same as (D) but for Ftz-F1 (Weirauch *et al*, 2014).

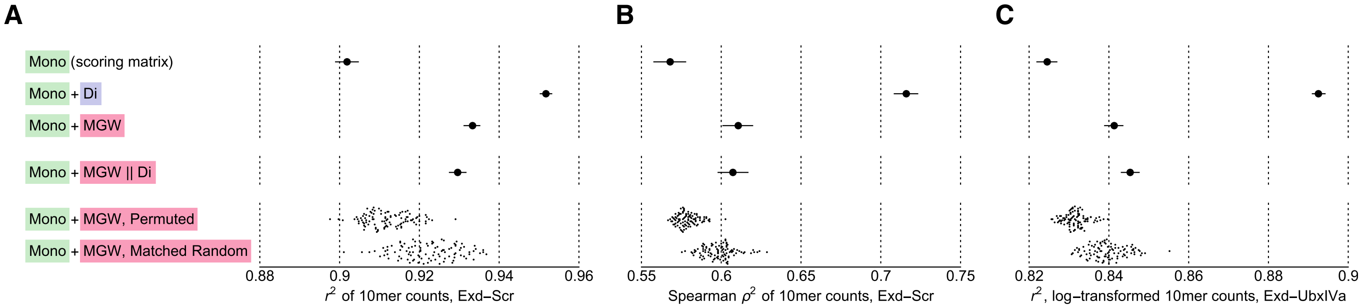

Figure EV2. Performance of direct shape regression models.

A Performance of Exd-Scr binding models computed as in Fig 4C–E except the performance was quantified using the Pearson correlation (r^2) between the 10mer counts instead of the log-transformed 10mer counts.

B The same as (A) but using the Spearman rank correlation coefficient (ρ^2).

C Performance of Exd-UbxIVa binding models computed as in Fig 4C–E.

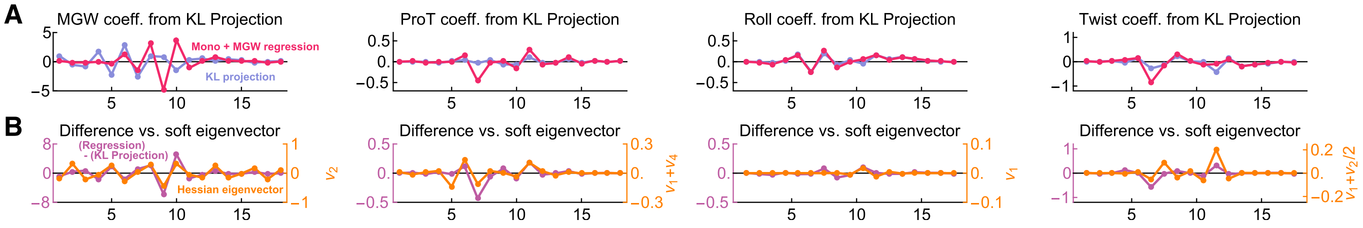

Figure EV3. Instabilities in inferred shape-sensitivity profiles correspond to small-eigenvalue ("soft") directions in the objective function.

A Shape-sensitivity coefficient from direct shape regression using NRLB (magenta) and from unpenalized shape projection based on a KL divergence loss function (blue).

B Comparison of the difference between the regression and the projection profiles in (A) (purple) and specific linear combinations of the eigenvectors v_i with the i-th smallest eigenvalues (orange).

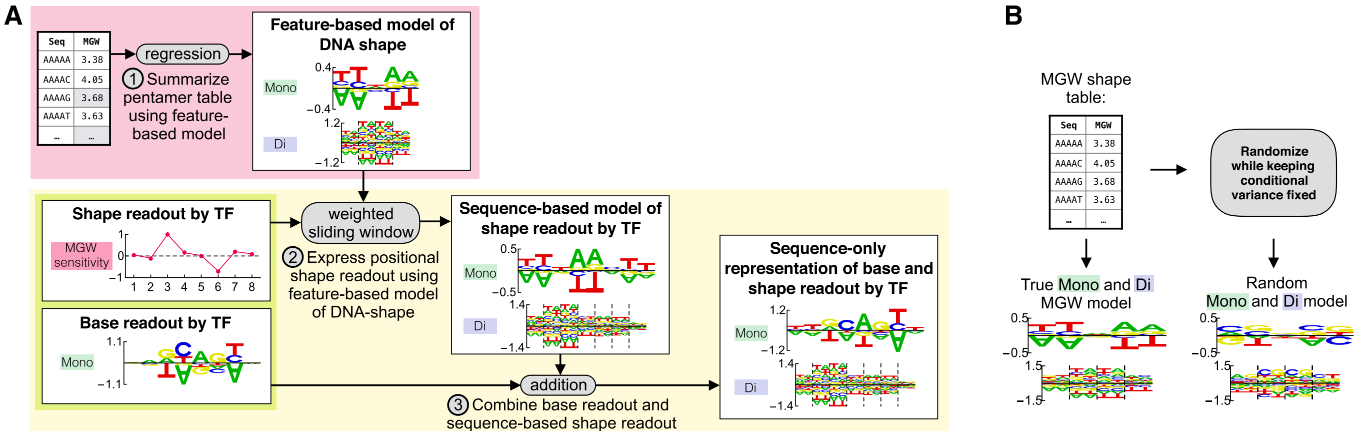

Figure EV4. Representing shape readout using sequence features alone.

A Schematic illustrating the procedure for expressing base and shape readout using sequence features alone.

B Illustration showing how random sequence-to-shape models were generated to match the conditional variance of the shape parameters after holding out pairs of bases.

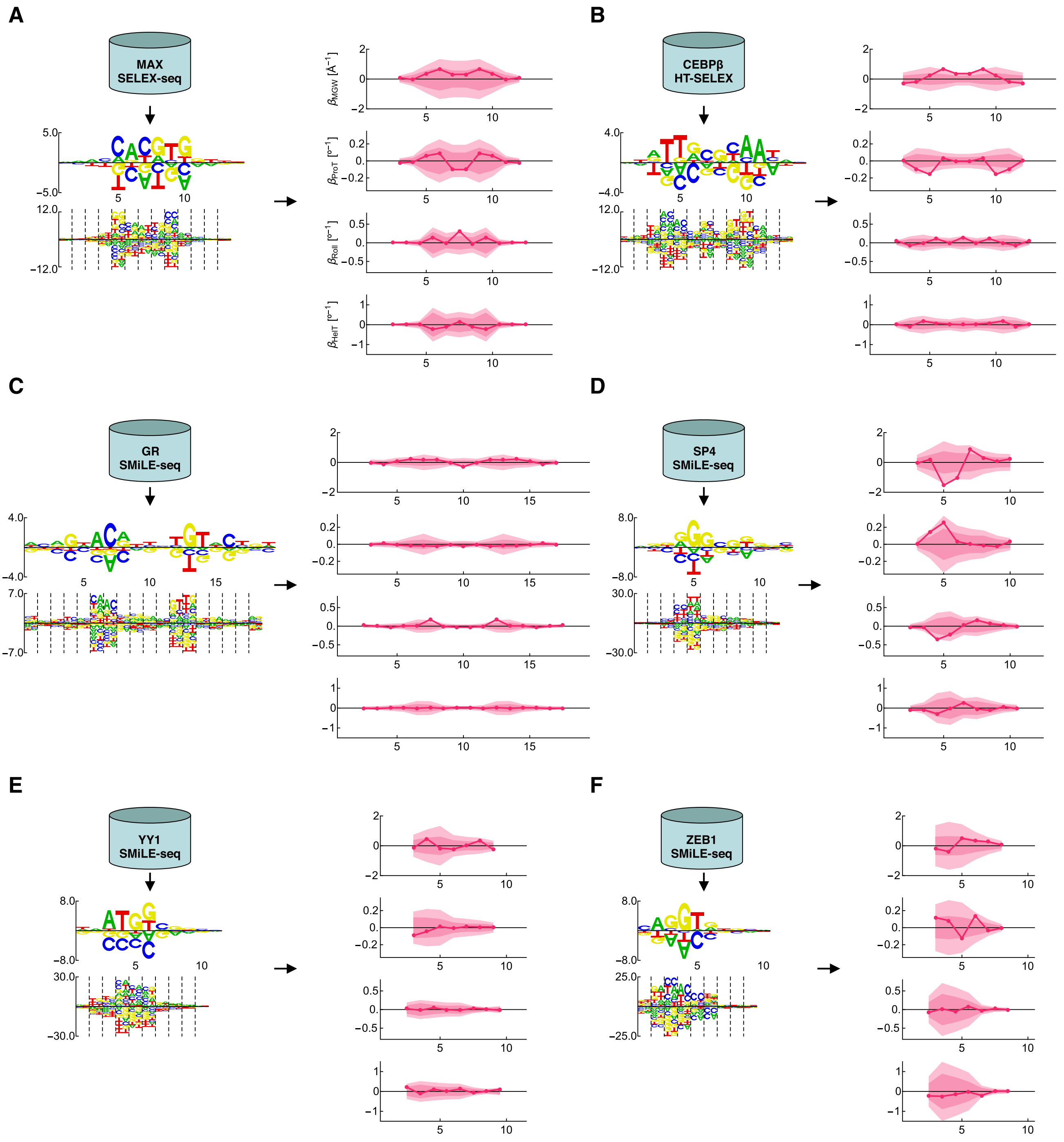

Figure EV5. Shape-sensitivity profiles computed using shape projection.

A Shape-sensitivity profiles computed from SELEX-seq data for the human factor MAX (Zhou *et al*, 2015), shown as in Fig 5.

B The same as (A) but using HT-SELEX data for human CEBPβ (Yang *et al*, 2017).

C–F The same as (A) but using SMiLE-seq data for the human nuclear hormone receptor GR, the murine factor SP4, the human zinc finger protein YY1, and the murine zinc finger protein ZEB1 (Isakova *et al*, 2017).

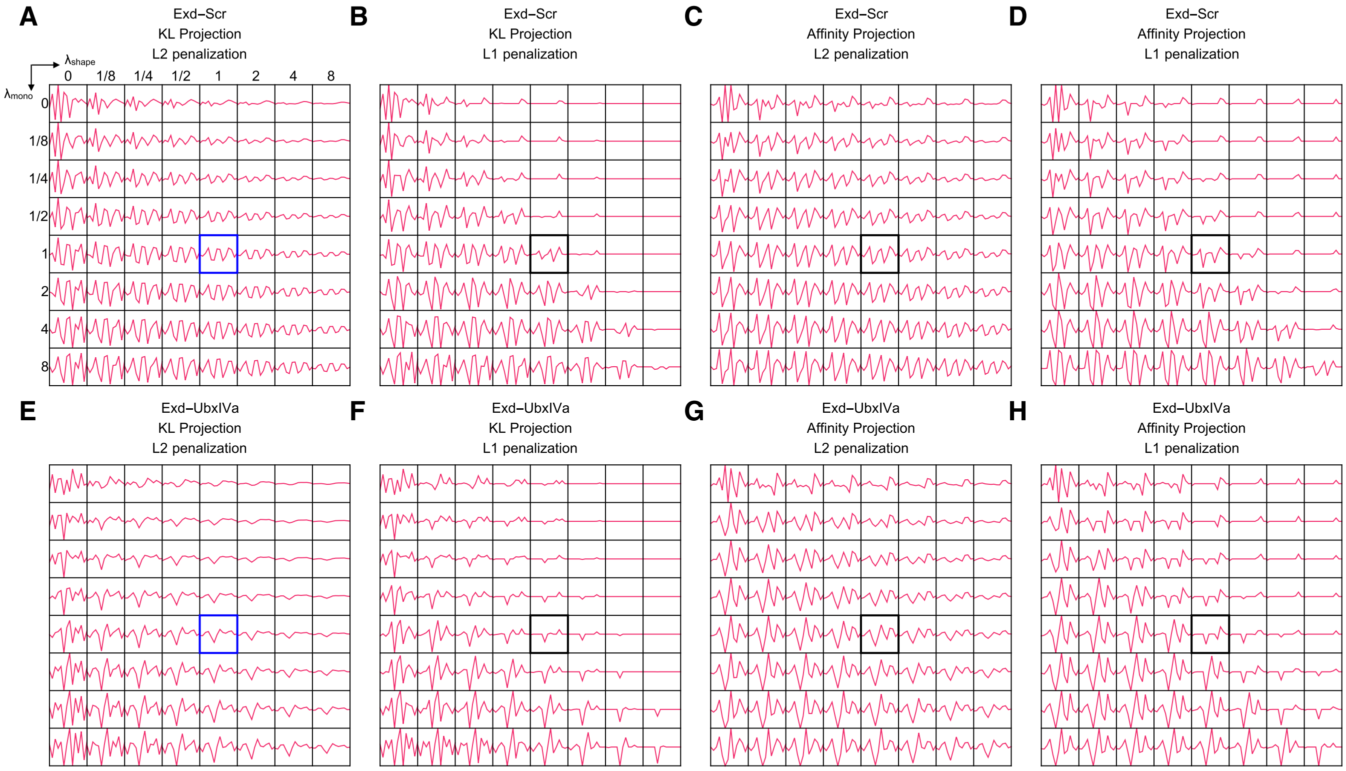

Figure EV6. Penalized shape projection using different loss functions and varying the scale parameter penalization term.

A MGW sensitivity coefficients for Exd-Scr inferred using L_2-penalized shape projection with KL divergence loss function. The penalization depends on the two normalized scale parameters λ_{mono} (which penalizes base readout) and λ_{shape} (which penalizes shape readout), both of which are set to unity by default. Each subplot shows the MGW sensitivity coefficients for one choice of these parameters.

B The same as (A) but using L_1 penalization.

C, D The same as (A, B) but using with the mean squared affinity error as loss function.

E–H The same as (A–D) but showing sensitivity profiles for Exd-UbxIVa. Squares with thick lines indicate the default parameter choice. Blue squares highlight the profiles shown in Fig 5B and C.
